# Severe portal and systemic acidosis during CO_2_-laparoscopy compared to helium or gasless laparoscopy and laparotomy in a rodent model: an experimental study

**DOI:** 10.1007/s00464-021-08810-6

**Published:** 2021-11-05

**Authors:** Devdas T. Inderbitzin, Tobias U. Mueller, Grischa Marti, Simone Eichenberger, Benoît Fellay, Jean-Luc Magnin, Lukas Kraehenbuehl

**Affiliations:** 1 Department of Cardiac Surgery, University Hospital of Zurich, Raemistrasse 100, 8091 Zurich, Switzerland; 2 Department of Anatomy, Faculty of Science and Medicine, University of Fribourg, 1700 Fribourg, Switzerland; 3 Department of Visceral and Transplantation Surgery, University Hospital of Zurich, Raemistrasse 100, 8091 Zurich, Switzerland; 4 Department of Internal Medicine, Hospital of Langenthal, 4900 Langenthal, Switzerland; 5 Department of Haematology and Clinical Chemistry, Hôpital Fribourgeois HFR, 1708 Fribourg, Switzerland; 6 Department of Surgery, Bauchzentrum Medical Center See-Spital, Gruetstrasse 55, 8802 Kilchberg, Zurich, Switzerland

**Keywords:** Laparoscopy, Blood gases, Acidosis, Insufflation gas, Insufflation pressure

## Abstract

**Background and aims:**

This experimental study assesses the influence of different gases and insufflation pressures on the portal, central-venous and peripheral-arterial pH during experimental laparoscopy.

**Methods:**

Firstly, 36 male WAG/Rij rats were randomized into six groups (*n* = 6) spontaneously breathing during anaesthesia: laparoscopy using carbon dioxide or helium at 6 and 12 mmHg, gasless laparoscopy and laparotomy. 45 and 90 min after setup, blood was sampled from the portal vein, vena cava and the common femoral artery with immediate blood gas analysis. Secondly, 12 animals were mechanically ventilated at physiological arterial pH during 90 min of laparotomy (*n* = 6) or carbon dioxide laparoscopy at 12 mmHg (*n* = 6) with respective blood gas analyses.

**Results:**

Over time, in spontaneously breathing rats, carbon dioxide laparoscopy caused significant insufflation pressure-dependent portal acidosis (pH at 6 mmHg, 6.99 [6.95–7.04] at 45 min and 6.95 [6.94–6.96] at 90 min, pH at 12 mmHg, 6.89 [6.82–6.90] at 45 min and 6.84 [6.81–6.87] at 90 min; *p* < 0.05) compared to laparotomy (portal pH 7.29 [7.23–7.30] at 45 min and 7.29 [7.20–7.30] at 90 min; *p* > 0.05). Central-venous and peripheral-arterial acidosis was significant but less severely reduced during carbon dioxide laparoscopy. Laparotomy, helium laparoscopy and gasless laparoscopy showed no comparable acidosis in all vessels. Portal and central-venous acidosis during carbon dioxide laparoscopy at 12 mmHg was not reversible by mechanical hyperventilation maintaining a physiological arterial pH (pH portal 6.85 [6.84–6.90] (*p* = 0.004), central-venous 6.93 [6.90–6.99] (*p* = 0.004), peripheral-arterial 7.29 [7.29–7.31] (*p* = 0.220) at 90 min; Wilcoxon–Mann–Whitney test).

**Conclusion:**

Carbon dioxide laparoscopy led to insufflation pressure-dependent severe portal and less severe central-venous acidosis not reversible by mechanical hyperventilation.

Laparoscopic interventions are performed since the late 1980s with steadily increasing numbers. Laparoscopy (LS) has become well accepted even for complex abdominal surgery as this minimally invasive technique has several advantages over laparotomy (LT) such as lower postoperative pain due to smaller incisions, shorter hospital stay and time to recovery [1, 2]. In clinical practice, LS is commonly performed establishing a carbon dioxide (CO_2_) pneumoperitoneum, as it is cheap and inert [3]. Its effect on cardiopulmonary [4–6], renal [5, 7–9] and metabolic functions [5] as well as on immunologic and inflammatory response [5, 10, 11] have broadly been investigated, but still little is known about the impact of CO_2_-LS on the liver function.

In vitro, CO_2_ leads to intracellular acidosis followed by impairment of mitochondrial function, suppression of immunologic response or increased tumour cell growth [11, 12]. Experimental in vivo models suggest an enhanced growth of colorectal hepatic metastases under exposure to CO_2_-LS compared to LT [13–15] or a decrease in portal blood flow during CO_2_-LS [16]. Clinical studies showed a systemic metabolic acidosis and provide an insight mainly into portal blood flow patterns, intraperitoneal acidosis and alteration of liver enzymes under CO_2_-LS [9, 17–19].

It is hypothesized that reabsorption of CO_2_ at the splanchnic peritoneum leads to systemic acidosis, causing acidosis in the portal vein with subsequent acidic liver flushing [9, 20, 21] potentially explaining several observed effects of CO_2_-LS in the liver [9, 12–14, 16]. Changes in liver function under experimental LS in a pig model were associated with alterations in portal-venous pH with a tendency towards acidosis compared to LT [12]. However, the effect of different gases and gasless LS as well as insufflation pressures were to the best of our knowledge so far not investigated.

This study aims to assess portal-venous, central-venous and peripheral-arterial blood gases by two experiments with experimental LS using CO_2_ and helium (He) insufflation at different insufflation pressures as well as gasless LS in comparison to LT, in a rodent model. Firstly, portal-venous, central-venous and peripheral-arterial pH alterations during exposure to LS with CO_2_ and He compared to gasless LS and LT were assessed to evaluate the influence of the gas-quality and insufflation pressure individually. Secondly, potential compensation of portal and systemic pH alterations by mechanical ventilation were assessed in this experimental setting.

## Materials and methods

### Animals

Forty-eight male WAG/Rij rats (Wistar-derived strain), weighing 295–370 g (Harlan, Horst, The Netherlands), were used for all experiments. They were kept under specific pathogen-free laboratory conditions with a controlled light and climate cycle and were fed a standard rat chow (Kliba Futter, Basel, Switzerland) with free access to sterile water. The animals were given a two weeks of adaptation period and fasted six hours before surgery. Experiments were approved by the local Animal Ethics Committee of the State of Fribourg, Switzerland, and performed according to international guidelines.

### Study design

First experiment: 36 rats were randomized into six operative groups (*n* = 6) with inhalation anaesthesia spontaneously breathing: CO_2_- and He-LS at 6 mmHg and 12 mmHg insufflating pressure each, gasless LS using abdominal wall retractors and LT (control group), respectively.

Second experiment: 12 rats were randomized into two operative groups (n = 6) with mechanically ventilated anaesthesia maintaining a physiological peripheral-arterial pH of 7.3–7.4 by adapting the tidal frequency: CO_2_-LS at 12 mmHg insufflating pressure and LT (control group).

**Fig. 1:**
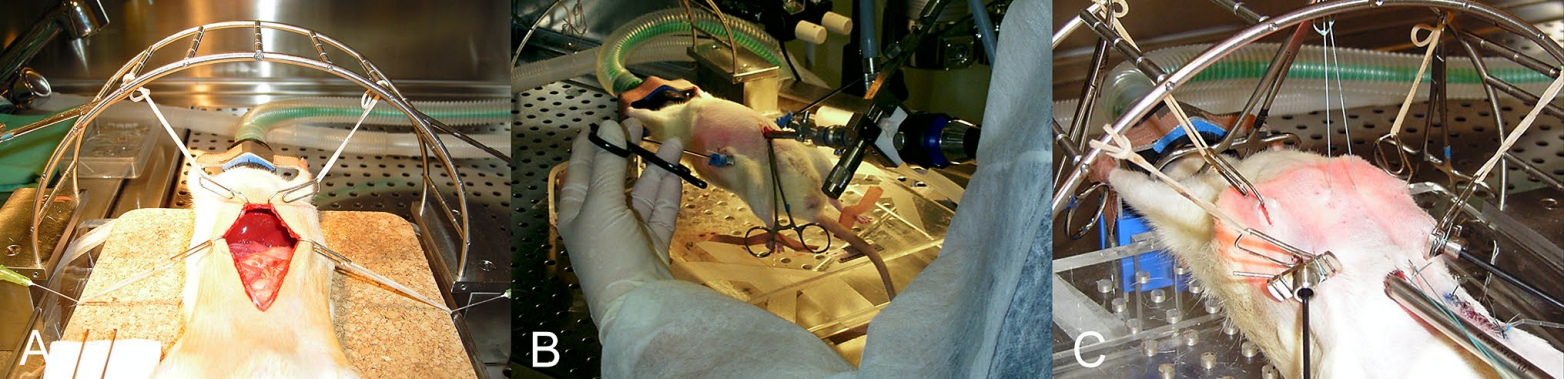
Experimental surgical set-up for laparotomy, laparoscopy and gasless laparoscopy. Operation techniques: **A** Laparotomy, **B** laparoscopy with pneumoperitoneum, **C** gasless laparoscopy with abdominal wall lift and atmospheric intra-abdominal pressure

**Fig. 2:**
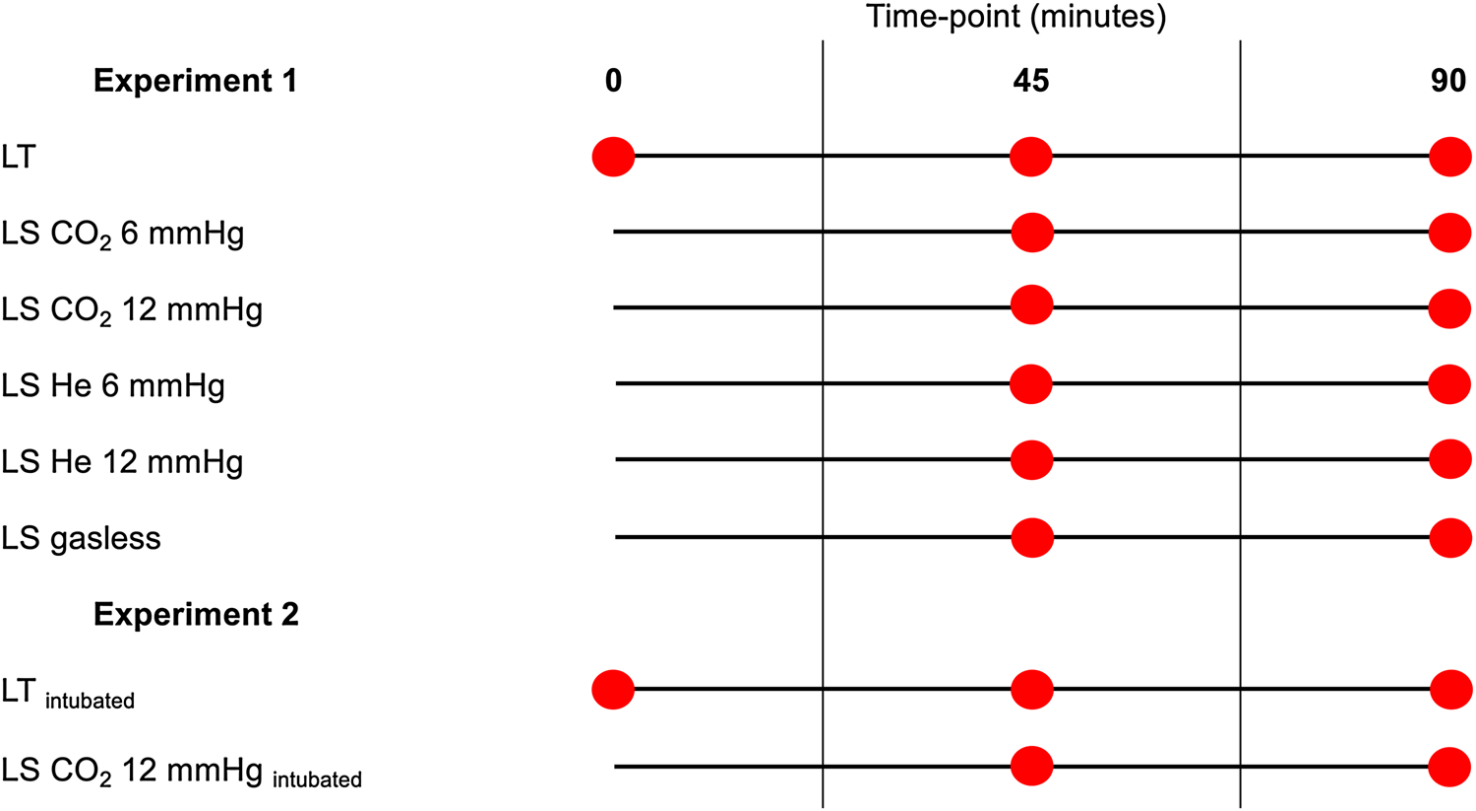
Illustration of surgical groups and time-points of blood sampling after setup: LT/LT intubated: laparotomy as control group. LS CO_2_ 6 mmHg: laparoscopy with CO_2_ insufflation at 6 mmHg. LS CO_2_ 12 mmHg/LS CO_2_ 12 mmHg intubated: laparoscopy with CO_2_ insufflation at 12 mmHg. LS He 6 mmHg: laparoscopy with helium insufflation at 6 mmHg. LS He 12 mmHg: laparoscopy with helium insufflation at 12 mmHg. LS gasless: gasless laparoscopy with abdominal wall lift at atmospheric intra-abdominal pressure

In both experiments, blood was sampled from the portal vein, vena cava and common femoral artery with immediate blood gas analysis at intra-operative time-points 0 (LT only), 45 and 90 min after surgical setup (all groups) (Figs. 1 and 2).

### Surgical procedures and anaesthesia

The animals were anaesthetized by inhalation of a mixture of isoflurane and oxygen (0.5–5% isoflurane complemented with pure oxygen) using a conventional vaporizer (Provet AG, Lyssach, Switzerland) after induction of narcosis in the induction-box. Spontaneously breathing animals in experiment 1 were vaporized by a table-fixed animal-mask (Provet AG, Lyssach, Switzerland), whereas animals in experiment 2 were intubated (16 or 18 G Venflon, Becton Dickinson GmbH, Heidelberg, Germany) and mechanically ventilated at a constant tidal volume of 1 ml/100 g and a dynamic tidal frequency (40–85 tidals/min) according to a referential peripheral-arterial pH of 7.3–7.4. The animals were then secured in a supine position on a small animal operating table and shaved subsequently. The overall operating time was 100 min including a 10 min time span for arterial catheter implantation prior to experimental surgery and measures. All animals were sacrificed after surgery.

For femoral catheter implantation prior to abdominal intervention, a cranio-caudal 1.5 cm subinguinal incision was followed by the blunt preparation of the left common femoral artery. After distal ligation and proximal clamping, the artery was cannulated using a 0.8 mm catheter (Polythene tubing; SIMS Portex, UK/LUER Lock adapter 0.7 mm OD). Successful flushing of the catheter with heparin was followed by fixation of the same and wound closure using a 3–0 Synthofil (B. Braun Medical SA, Emmenbrücke, Switzerland) running suture in two layers. The stitches were close (3 mm) and tight enough to prevent bleeding and gas-leakage. Flushing solution consisted of Heparin 50 UI/ml (Sanofi-Synthelabo SA, Meyrin, Switzerland) in saline (0.9% NaCl).

Pneumoperitoneum was provided by a CO_2_-insufflator (Aesculap, Tuttlingen, Germany) or an adapted Helium-insufflator (Endoflator by Karl Storz Endoskope, Tuttlingen, Germany) insufflating at pressures of 6 and 12 mmHg, respectively. To avoid intra-abdominal pressure fluctuation due to instrument- and trocar-sleeve-movements, a 5-l rigid metal gas tank was placed between insufflator and the rodent’s abdominal cavity. Hence, the resulting intra-abdominal pressure was kept constant by simulation of human abdominal volume dimensions. Gas flow was below 100 ml/min but not measured in detail. LS was performed using a 30° 4 mm arthroscope with a 4.5 mm trocar sleeve (Aesculap, Tuttlingen, Germany) and two 3 mm trocars (working channels) and 2.7 mm instruments (scissors, straight forceps; Wolf, Tuttlingen, Germany). Ordinary microsurgical instruments were used for open procedures. During gasless LS and LT (standardized 5 cm midline incision), the abdominal wall was retracted by metal hooks (made of ordinary paperclips) and rubber-bands fixed to a metal arch and a cork-mat. Retractors in gasless LS were placed in the midline, 1 cm below the xyphoid, and at both sides laterally as well as at the trocar sleeves of the working channels. The abdominal cavity was protected with moistened sterile gauzes during the absence of open surgical manipulation.

Intra-abdominal blood sampling began with the blunt exposition of the portal vein and the vena cava using 5 mm cotton swabs to move the intestines. There were no intra-abdominal adhesions and no exposure-associated bleeding occurred. The vessels were approached from behind the duodenal loop. Prior to vessel puncture, 4 cotton swabs were placed on the liver surface and kept near and ready to stop eventual bleeding. A 12-cm-long catheter (needle and adaptor: LUER Lock 0.4 × 12 mm, silicone tubing 0.6 mm OD; Ulrich AG, St. Gallen, Switzerland) was introduced into abdominal cavity by a 2 mm abdominal wall incision at the right lower quadrant. The incision was thereafter clamped to avoid gas-leakage. At 45 and 90 min after setup, blood sampling was provided by sequential puncture of the portal vein and the vena cava and through the femoral arterial catheter. The open sampling included a supplementary analysis immediately after LT and was performed using the same needle-armed catheter and cotton swabs. In the abdominal vessels, the needle was retracted and flushed with 0.9% NaCl after sampling and bleeding was avoided by gentle pressure of a cotton swab onto the puncture site for 2 min. The blood loss after retraction of the needle from the puncture sites was minimal and therefore negligible. The retracted blood volume, 250 µl for each sample, was replaced by 0.9% NaCl through the femoral catheter. Total blood loss for complete sampling was 2.25 ml and 1.5 ml per animal in the LT- and LS groups, respectively. The collected samples were processed immediately after collection. One collective sampling took no longer than 8 min.

### Processing of blood samples and blood gas analysis

Each sample was collected in a 1 ml syringe (B. Braun Medical SA, Emmenbrücke, Switzerland) through a 12-cm-long catheter (needle and adaptor: LUER Lock 0.4 × 12 mm, silicone tubing 0.6 mm OD) and processed without delay. The blood was transferred into two (sample plus spare) heparinized capillary tubes (115 µl; AVL, Roche Diagnostics, Switzerland), hermetically closed with two capillary caps, mixed using a magnetic stick, labelled, kept in a thermostable obscure box and transferred to the nearby laboratory of the cantonal hospital of Fribourg for blood gas analysis within 15 min (Radiometer ABL 700, Diamond Diagnostics, MA, USA).

### Data analysis

Both endpoints, pH (primary) and pCO_2_ (secondary), were analysed the same way except that pCO_2_ was log-transformed to achieve normal distribution. The pH assessed at all three vessels (portal vein, vena cava, common femoral artery) was analysed overtime for the operation groups (LT for time-points 0, 45 and 90 min and for all LS groups for time-points 45 and 90 min). In this study, inter- and intragroup pH differences were observed overtime. Due to logistic and surgical reasons, it was not possible to acquire a baseline pH value for time-point 0 min in all LS groups. At comparable animal groups (age, sex, weight) and equal environmental conditions at the operation room, the pH and pCO_2_ on time-point 0 min during LT were extrapolated as baseline pH for all groups.

To evaluate the impact of pressure on pH intergroup differences for each gas type (CO_2_, He) at time-points 45 and 90 min for all three vessels (portal vein, vena cava, common femoral artery) and to calculate the independent impact of gas and pressure, intubation, localization of measurement and effect of time on the endpoints, all comparisons between groups were conducted non-parametrically, using the Wilcoxon–Mann–Whitney test. The data are presented as median with interquartile range (IQR).

Significance level (alpha) was set to < 0.05 for all analyses. Statistical procedures were performed using Stata 14.2 (Stata Corp. 2015, College Station, TX).

## Results

### Animals

The mean body weight of all male rats at the point of intervention was 310 g [306–318] in animals undergoing laparotomy and 325 g [315–334] in the group of laparoscopies. There was no significant difference between the surgical groups in body weight or age (*p* = 1.00).

### Experiment 1: intergroup pH differences

Animals exposed to pneumoperitoneum with CO_2_ or He developed portal, central-venous and peripheral-arterial (common femoral artery) acidosis over 90 min of exposure with a tendency to aggravate over time (Table 1). For all LS groups with gas insufflation, there was a significant acidosis over time in all vessels compared to the LT group (*p* < 0.03) in spontaneously breathing animals (except for the He-LS 6 mmHg at time-points 45 min after setup for the vena cava and the common femoral artery, and at time-point 90 min after setup for the vena cava and the portal vein (*p* > 0.05)). In gasless LS, there was no significant acidosis over time in all vessels compared to the LT group (*p* > 0.05) in spontaneously breathing animals. However, central-venous and peripheral-arterial acidosis was significant but less severe compared to the portal acidosis during CO_2_-LS. In contrast, LT, He-LS and gasless LS showed no comparable acidosis compared to CO_2_-LS in all vessels.

**Table 1:** Experiment 1: Blood gas analysis: pH in portal vein, vena cava and common femoral artery over time in spontaneously breathing animals

Group	Location	Time-point (min)			*p* for 0 to 45	*p* for 45 to 90	*p* for 0 to 90
		0	45	90			
LT	Portal	7.32 [7.26–7.35]^a^	7.29 [7.23–7.30]	7.29 [7.20–7.30]	0.200	0.870	0.200
	Femoral	7.31 [7.28–7.33]^a^	7.30 [7.27–7.32]	7.29 [7.24–7.30]	0.520	0.340	0.200
	Caval	7.32 [7.29–7.36]^a^	7.29 [7.26–7.31]	7.28 [7.26–7.30]	0.230	0.520	0.080
CO_2_-LS 6	Portal	–	6.99 [6.95–7.04]	6.95 [6.94–6.96]	0.004*	0.170	0.004*
	Femoral	–	7.16 [7.15–7.17]	7.11 [7.09–7.12]	0.004*	0.030*	0.004*
	Caval	–	7.05 [6.95–7.09]	6.96 [6.91–7.01]	0.004*	0.050	0.004*
CO_2_-LS 12	Portal	–	6.89 [6.82–6.90]	6.84 [6.81–6.87]	0.004*	0.200	0.004*
	Femoral	–	7.01 [6.95–7.03]	6.94 [6.92–6.99]	0.004*	0.080	0.004*
	Caval	–	6.86 [6.85–6.89]	6.83 [6.81–6.86]	0.004*	0.340	0.004*
He-LS 6	Portal	–	7.22 [7.21–7.27]	7.25 [7.23–7.26]	0.025*	0.420	0.050
	Femoral	–	7.24 [7.20–7.26]	7.24 [7.21–7.27]	0.006*	0.870	0.025*
	Caval	–	7.26 [7.23–7.28]	7.23 [7.20–7.26]	0.010*	0.340	0.010*
He-LS 12	Portal	–	7.17 [7.15–7.20]	7.16 [7.12–7.16]	0.004*	0.200	0.004*
	Femoral	–	7.16 [7.14–7.19]	7.16 [7.13–7.16]	0.004*	0.340	0.004*
	Caval	–	7.19 [7.17–7.20]	7.16 [7.14–7.18]	0.004*	0.110	0.004*
Gasless LS	Portal	–	7.22 [7.20–7.24]	7.25 [7.18–7.30]	0.037*	0.870	0.230
	Femoral	–	7.23 [7.22–7.26]	7.29 [7.17–7.30]	0.050	0.750	0.200
	Caval	–	7.26 [7.24–7.26]	7.22 [7.18–7.27]	0.025*	0.200	0.025*

pH (median ± interquartile range) at 0, 45, 90 min after setup of laparotomy (LT)/laparoscopy (LS) at 6 and 12 mmHg (LS 6 or LS 12); portal vein (portal), vena cava (caval), common femoral artery (femoral) by spontaneously breathing animals. Intergroup differences between pH are significant between LT and all LS groups (*p* < 0.05), except between gasless LS in all vessels and He-LS 6 mmHg for the vena cava and common femoral artery at time-point 45 min and for the vena cava and the portal vein at time-point 90 min after setup (*p* > 0.05) (Mann–Whitney *U* test)

*Reached level of significance

^a^Measured results at time-point 0 min in LT group were used as referring baseline for all other surgical groups

### Experiment 1: intragroup pH alteration over time

In the CO_2_-LS group, a highly significant pH-drop with a tendency to aggravate over time was detected in all vessels as shown in Table 1. In the portal vein, the lowest pH for CO_2_-LS 6 mmHg was measured after 90 min with 6.95 [6.94–6.96] (*p* = 0.004) and for CO_2_-LS 12 mmHg after 90 min with 6.84 [6.81–6.87] (*p* = 0.004). The portal, central-venous and peripheral-arterial pH-drop was significantly higher at a higher insufflation pressure (Table 2).

**Table 2:** Experiment 1: blood gas analysis: pH differences between the operation groups in spontaneously breathing animals

Location	Time-point (min)	CO_2_-LS 6	CO_2_-LS 12	*p*-value	He-LS 6	He-LS 12	*p*-value
Portal	45	6.99 [6.95–7.04]	6.89 [6.82–6.90]	0.010*	7.22 [7.21–7.27]	7.17 [7.15–7.20]	0.050
	90	6.95 [6.94–6.96]	6.84 [6.81–6.87]	0.006*	7.25 [7.23–7.26]	7.16 [7.12–7.16]	0.024*
Femoral	45	7.16 [7.15–7.17]	7.01 [6.95–7.03]	0.004*	7.24 [7.20–7.26]	7.16 [7.14–7.19]	0.024*
	90	7.11 [7.09–7.12]	6.94 [6.92–6.99]	0.004*	7.24 [7.21–7.27]	7.16 [7.13–7.16]	0.025*
Caval	45	7.05 [6.95–7.09]	6.86 [6.85–6.89]	0.010*	7.26 [7.23–7.28]	7.19 [7.17–7.20]	0.010*
	90	6.96 [6.91–7.01]	6.83 [6.81–6.86]	0.004*	7.23 [7.20–7.26]	7.16 [7.14–7.18]	0.025*

pH (median ± interquartile range) 45, 90 min after setup of LS with CO_2_ at 6 and 12 mmHg insufflation pressure (CO_2_-LS 6, CO_2_-LS 12) and of LS with helium at 6 and 12 mmHg insufflation pressure (He-LS 6, He-LS 12); portal vein (portal), vena cava (caval), common femoral artery (femoral) by spontaneously breathing animals. (Mann–Whitney *U* test)

*Reached level of significance

In the He-LS group, at a lower insufflation pressure, the portal pH change over time was not significant (portal pH during He-LS 6 mmHg after 90 min: 7.25 [7.23–7.26] (*p* = 0.050)). At a higher insufflation pressure, the portal, central-venous and peripheral-arterial acidosis was highly significant (*p* = 0.004), but not as pronounced as in the CO_2_-LS group (Table 1). The pH-drop was significantly higher with a high insufflation pressure, except in the portal vein at 45 min after setup (Table 2).

However, the effect of insufflation pressure is not as high as the impact of the gas-quality. All results for the CO_2_-LS and He-LS groups resulted after Wilcoxon–Mann–Whitney test with reference to the value at time-point 0 min from the LT group assuming similar starting values in the LS groups prior to LS-setup.

In the gasless LS, the central-venous acidosis became significant over time (pH central-venous gasless LS after 90 min: 7.22 [7.18–7.27] (*p* = 0.025), the portal vein and common femoral artery showed a non-significant tendency for acidosis, Table 1).

The differences of portal pH between the CO_2_ and He group at a given time-point and high insufflation pressure were highly significant (*p* = 0.004). This tendency was less within the non-CO_2_ groups (He, gasless LS and LT).

### Experiment 2: hyperventilation

In hyperventilated animals, there was no systemic or portal acidosis detected in the LT group. However, in the CO_2_-LS group, a highly significant portal and central-venous acidosis developed over time (pH portal after 90 min during CO_2_-LS 12 mmHg: 6.85 [6.84–6.90] (*p* = 0.004), pH central-venous after 90 min during CO_2_-LS 12 mmHg: 6.93 [6.90–6.99] (*p* = 0.004)). No significant peripheral-arterial pH alteration was measured during CO_2_-LS 12 mmHg (pH peripheral artery after 90 min during CO_2_-LS 12 mmHg: 7.29 [7.29–7.31] (*p* = 0.090)), except between the time-points 0 and 45 min (Table 3).

**Table 3:** Experiment 2: blood gas analysis: Alteration of pH in mechanically hyperventilated animals over time

Group	Location	Time-point (min)			*p* for 0 to 45	*p* for 45 to 90	*p* for 0 to 90
		0	45	90			
LT	Portal	7.32 [7.30–7.35]^a^	7.32 [7.28–7.33]	7.30 [7.26–7.31]	0.740	0.260	0.190
	Femoral	7.32 [7.29–7.34]^a^	7.36 [7.32–7.40]	7.34 [7.29–7.36]	0.060	0.300	0.480
	Caval	7.32 [7.28–7.36]^a^	7.28 [7.25–7.31]	7.32 [7.28–7.36]	0.110	0.130	0.740
CO_2_-LS 12	Portal	–	6.93 [6.91–7.00]	6.85 [6.84–6.90]	0.004*	0.200	0.004*
	Femoral	–	7.27 [7.25–7.30]	7.29 [7.29–7.31]	0.007*	0.170	0.090
	Caval	–	6.93 [6.84–6.97]	6.93 [6.90–6.99]	0.004*	0.810	0.004*

pH (median ± interquartile range) 0, 45, 90 min after setup of laparotomy (LT)/ laparoscopy (LS) with CO_2_ at 12 mmHg (CO_2_-LS 12); portal vein (portal), vena cava (caval), common femoral artery (femoral) by mechanically ventilated animals. In between group differences over time were highly significant for the portal vein and vena cava (*p* = 0.004), for the common femoral artery only at 45 min (*p* = 0.007) but not at 90 min (*p* = 0.170) (Mann–Whitney *U* test)

*Reached level of significance

^a^Measured results at time-point 0 min in LT group were used as referring baseline for all other surgical groups

Over time there was no significant peripheral-arterial pH alteration neither in the LT nor CO_2_-LS group. On the other hand, there was a significant pH difference between the two groups in the portal vein at time-points 45 and 90 min after set-up (Table 4).

**Table 4:** Experiment 2: blood gas analysis: pH differences between the operation groups in mechanically hyperventilated animals

Location	Time-point (min)	LT	CO_2_-LS 12 mmHg	*p*-value
Portal	45	7.32 [7.28–7.33]	6.93 [6.91–7.00]	0.004*
	90	7.30 [7.26–7.31]	6.85 [6.84–6.90]	0.004*
Femoral	45	7.36 [7.32–7.40]	7.27 [7.25–7.30]	0.008*
	90	7.34 [7.29–7.36]	7.29 [7.29–7.31]	0.220
Caval	45	7.28 [7.25–7.31]	6.93 [6.84–6.97]	0.004*
	90	7.32 [7.28–7.36]	6.93 [6.90–6.99]	0.004*

pH (median ± interquartile range) 45, 90 min after setup of laparotomy (LT)/laparoscopy (LS) with CO_2_ at 12 mmHg (CO_2_-LS 12); portal vein (portal), vena cava (caval), common femoral artery (femoral) by mechanically ventilated animals. (Mann–Whitney *U* test)

*Reached level of significance

Over the time of 45 and 90 min of CO_2_-LS at 12 mmHg significant portal acidosis developed whilst peripheral-arterial pH was kept normal by means of mechanical hyperventilation (portal pH 6.93 [6.91–7.00] at 45 min and femoral pH 7.27 [7.25–7.30] at 45 min during CO_2_-LS 12 mmHg (*p* = 0.004); portal pH 6.85 [6.84–6.90] at 90 min and femoral pH 7.29 [7.29–7.31] at 90 min during CO_2_-LS 12 mmHg; *p* = 0.004). No significant intragroup pH difference between the portal vein and the common femoral artery was observed at any time-point during LT.

The analysis for pCO_2_ reflected the pH findings and is therefore not further described or discussed in this study.

## Discussion

This experimental study reports that CO_2_ pneumoperitoneum led to a significant acidosis in the portal vein, vena cava and common femoral artery compared to LT, helium or gasless LS with a tendency to aggravate under increased insufflation pressure and over time in spontaneously breathing rats. Hereby, the observed influence of the insufflation pressure compared to the gas-quality on the portal and systemic pH-drop was minimal. Furthermore, there was no correction of the portal and central-venous acidosis by means of mechanical ventilation. An overview of the main findings in context with the current state of literature is summarized in Tables 5 and 6. Due to ethical considerations, the number of experimented animals was limited in all groups. Hence, the results and outcome of the present study provide indications rather than definite results with regard to the influence of CO_2_ on portal and systemic blood gases. Larger and more experimental and clinical studies are needed to confirm the presented findings.

**Table 5:** Summary of main findings and their context to the current state of literature. Experiment 1: intragroup differences focussed on the portal vein

Group	Main finding	Findings in comparison to current state of literature
Laparotomy	No significant acidosis or hypercapnia over time	The results support the findings of Yoshida et. al. as they reported no change in portal pH during laparotomy [12]
CO_2_-laparoscopy 6 mmHg	Significant acidosis and hypercapnia over time and compared to LT, but no significant change between 45 and 90 min	These findings confirm the known transperitoneal diffusion of CO_2_ during laparoscopy with subsequent systemic acidosis and the single previously measured acidosis in the portal vein in an experimental animal model [5, 12, 20–22]
CO_2_-laparoscopy 12 mmHg	Significant acidosis and hypercapnia over time and compared to LT, but no significant change between 45 and 90 min	In addition to the CO_2_-laparoscopy group with 6 mmHg, the pH decrease is higher in absolute numbers and hence more accentuated. This supports the knowledge that the acidosis is mainly dependent on the gas-quality with an aggravation under higher insufflation pressure due to an increased mechanical ventilatory restriction. The pH of the portal vein was to the best of our knowledge not measured under different insufflation pressures. However, the influence of an increased insufflation pressure on the systemic pH was shown before [5, 24]
He-laparoscopy 6 mmHg	No significant acidosis or hypercapnia over time, except between time-point 0 and 45 min	These findings support the previously described absence of significant acidosis or hypercapnia during helium laparoscopy [30]. The initial significant pH change is thought to be triggered by mechanical ventilatory restriction during set-up of the pneumoperitoneum [5]. However, the pH of the portal vein was to the best of our knowledge not measured during experimental He-laparoscopy
He-laparoscopy 12 mmHg	Significant acidosis and hypercapnia over time, but not as pronounced as in the CO_2_ groups	These findings support the previously described mechanical ventilatory restriction due to a high intra-abdominal insufflation pressure that is responsible for the significant acidosis in spontaneously breathing animals [5, 24, 30]. However, as mentioned for the He-laparoscopy group with 6 mmHg, no measurement of the portal pH has been published so far during experimental He-laparoscopy
Gasless laparoscopy	No significant acidosis or hypercapnia over time	No changes in systemic pH are described in the current literature [6, 33]. However, the pH in the portal vein has not been measured yet during gasless laparoscopy

Summary of the results in the portal vein for experiment 1 in context with the existing literature. The literature is cited in the reference list at the end of the article

*CO*_*2*_ carbon dioxide, *He* helium

**Table 6:** Summary of main findings and their context to the current state of literature. Experiment 2: hyperventilation in CO_2_-laparoscopy compared to laparotomy

Group	Main finding	Significance in comparison to current state of literature
Laparotomy intubated	No significant systemic or portal acidosis over time. No significant intragroup differences between the portal vein and the common femoral artery	No changes in portal or peripheral pH during laparotomy were also shown by Yoshida et al. [12]
CO_2_-laparoscopy 12 mmHg intubated	Significant portal acidosis and hypercapnia over time and a significant pH difference between the portal vein and the common femoral artery. No significant change of the peripheral-arterial pH over time	These findings support the current knowledge about the transperitoneal diffusion of CO_2_ during laparoscopy and the achievable correction of the systemic acidosis by hyperventilation [5, 12, 20–22, 25]. New findings are that there is no correction of portal acidosis by mean of hyperventilation during experimental laparoscopy with CO_2_

Summary of the results of experiment 2 in context with the existing literature. The literature is cited in the reference list at the end of the article

*CO*_*2*_ carbon dioxide

The portal vein drains blood from the splanchnic circulation towards the liver including blood from the peritoneal capillary system. A rise of systemic CO_2_-partial pressure during CO_2_-LS in humans suggests a potential splanchnic CO_2_-absorption during pneumoperitoneum [20, 22]. Although, trans-peritoneal transition of gas-molecules into the tissue demonstrated the evidence of absorption into splanchnic and portal blood flow is still lacking [23]. Systemic acidosis can be corrected by means of hyperventilation [20], whereas portal acidosis might not be correctable prior to subsequent acidic liver perfusion. To the current state of literature, portal pH has so far been measured during CO_2_-LS in a porcine model, without comparing different insufflation pressures or gas-qualities or gasless LS [12].

This study’s findings support our hypothesis that the portal vein directs acidic blood from the splanchnic circulation towards the liver during experimental CO_2_-LS as CO_2_ pneumoperitoneum caused significant portal-venous acidosis tending to aggravate under increased insufflation pressure and over time. Considering the cited systemic effect of CO_2_ pneumoperitoneum, similar pH alterations were observed in the other examined vessels correlating to the insufflation pressure [5, 9, 20, 22]. During He-LS in spontaneously breathing animals, a tendency towards falling pH values in all vessels was registered but pH changes only reached significance at a high insufflation pressure. This suggests a direct mechanical effect of the insufflation pressure by mechanical ventilatory restriction affecting portal and systemic blood gases [5]. This could relativize the gas-quality-dependent pH-drop during CO_2_-LS and the pressure-dependent mechanical disturbance of spontaneous ventilation, peritoneal micro-circulation or portal-venous flow, as it was described by Junghans et al. [24], as a reasonable explanation for this effect.

Hence, we postulate a marginal impact of the insufflation pressure on the portal pH, as acidic pH alteration turned significant only during CO_2_-LS 6 mmHg but not during He-LS 6 mmHg, indicating the possibly higher impact of gas-quality over insufflation pressure [21, 25]. However, this study did not investigate the portal-venous flow nor the reversibility of pH change through hyperventilation.

An important question remains whether portal acidosis is subject to hyperventilatory compensation. Therefore, the second experiment was carried out with 12 animals intubated and mechanically ventilated throughout CO_2_-LS to rule out potential errors caused by spontaneous breathing (due to ethical and logistic reasons only at high-pressure CO_2_-LS 12 mmHg). Hyperventilation was counter-checked by peripheral-arterial blood gas analysis to ensure constant peripheral-arterial pH-correction. According to our hypothesis, the portal and central-venous acidosis during CO_2_-LS could not be corrected by means of mechanical hyperventilation, whilst peripheral-arterial acidosis was completely reversed. These findings allow to hypothesize that portal acidosis is probable to occur during CO_2_-LS also in humans despite the anaesthesiologic concern to correct systemic acidosis by mechanical hyperventilation and to monitor it by peripheral blood gas analysis. Portal acidosis during CO_2_-LS as revealed in this study has not yet been measured in a clinical setting.

So far, no measurements of the portal pH in humans are published, most probably due to technical difficulties and related safety and ethical aspects. Yoshida et al. measured portal acidosis but did not investigate the effect of hyperventilation in a porcine model [12]. Furthermore, Hanly et al. reported a persistent local acidosis of the peritoneum during CO_2_-LS despite simultaneous respiratory correction of systemic acidosis [25]. These results may indicate the importance of potential hepatic acidosis under CO_2_-LS that may occur also in humans. The circumstance of CO_2_-LS leading to portal acidosis that cannot be corrected by hyperventilation could play a role in hepatic immunological and metabolic reactions. For example, a potential impairment of the liver function, as it was indicated by Yoshida et al. [12] in a porcine model, could be taken into consideration when performing abdominal tumour surgery, as there might be an impact on tumour cell growth and spreading of hepatic neoplasia [13–15, 26] given a constant acidic flushing of the liver during LS. However, it is unknown if these alterations also occur in humans.

The effect of acidosis within hepatic tissue caused by peritoneal CO_2_-absorption and acidic portal flushing on hepatic metabolism are still unknown in detail [9, 12], but might aggravate the course of pre-existing hepatopathy [9, 27]. However, the effect of hepatic tumour cell growth as a consequence of exposure to CO_2_-LS was already investigated previously and discussed in several studies [13, 15, 28, 29]. We tried to address this question in WAG rats developing colorectal cancer metastases in the liver subjected to CO_2_-LS. Using the same rat breed and equal experimental setup, an increased tumour growth and suppressed anti-tumoural cellular defence were found after direct subcapsular hepatic inoculation of colorectal tumour cells under CO_2_-LS compared to LT [14]. Considering the identical operative setup of the present and preceding tumour-study, their comparison might rise the suggestion of a certain correlation between CO_2_-LS with its associated portal acidosis and an enhanced hepatic colorectal tumour growth, that might be triggered by the potential mechanism of immunosuppression. However, further evidence for direct causality first needs to be demonstrated yet by a joint study.

As a cause of thought, considering the described side effects of CO_2_ pneumoperitoneum in patients undergoing prolonged surgery (e.g. abdominal tumour resection), helium could be considered as an alternative gas as described before [3, 13, 30, 31]. Despite the absence of evidence for fatal gas embolism or life-threatening complications (Cochrane Review [3]), the potential risk for gas embolization remains a concern regarding the higher solubility of helium. Due to the level of technical realization and the higher cost of helium, it did not breakthrough as a routine setup for abdominal surgery. Alternatively, abdominal wall lift at normal atmospheric pressure could account for another low risk alternative for liver surgery [32, 33].

Strengths of the present study are the careful operative setup with a 5-l rigid metal gas tank between the insufflator and the rodent’s abdominal cavity to provide a stable intra-abdominal pressure curve avoiding pressure fluctuations and subsequent irregular insufflating flow patterns as generated by the insufflator during LS. An equilibrated intra-abdominal pressure accounted for most possible stability of laparoscopic setup in a rat’s abdominal cavity by an insufflator designed for humans. Furthermore, additional attempts were undertaken to minimize potential errors owed to respiratory effects of spontaneously breathing animals by additional measures in mechanically hyperventilated animals.

Potential sources of error remain the delay of sampling until analysis, leaking of the hermetically sealed probes and potential aggravation of acidosis by spontaneously breathing animals (although peripheral-arterial measures excluded severe errors). A weakness of this study is the lack of measured pH values at time-point 0 min for the LS groups due to impaired access to the portal vein at the beginning of LS and the data-extrapolation of LT group to the LS group at time-point 0 min for data analysis. Moreover, the reversibility of pH changes by means of hyperventilation in the portal vein was not measured. The relatively small number of animals might even increase the margin of error of this study. Moreover, the use of a laparoscopic insufflator designed for humans did not allow exact recordings of flow-rates in the low range of a rat’s abdominal cavity. Hence, the gas-flow was neither limited nor recorded but estimated to be below 100 ml/min as to the insufflators’ alarms. Although intra-abdominal pressure profile was kept constant by a rigid 5-l metal equilibrating gas tank, the lack of gas-flow-measurement theoretically does not exclude supra-physiological rates of insufflation, what weakens a direct translation of this rodent model to a human setup.

Substitution of blood loss by 0.9% NaCl and its consequences on blood gas measurements are another point of concern. The blood loss by collective sample was 750 µl and hence 1.5 and 2.25 ml of blood in total for LS and LT, respectively. This acquaints for approximately 5% of the total blood volume of a rat (54–70 ml/kg). Therefore, we consider a significant influence of the substituted blood volume on the pH rather unlikely, though. Another relatively weak factor of error was the minimal blood loss when retracting the puncture needle from the portal or cava vein, as the cotton swabs were already placed in the intra-abdominal cavity prior to puncture and therefore were ready for immediate compression of the puncture site. Accordingly, the blood loss was minimal and negligible during this study, despite not nil and not substituted.

We conclude that CO_2_-LS leads to a significant portal acidosis and the liver flushing with acidic blood (pH < 7.0) during CO_2_ pneumoperitoneum which cannot be compensated by mechanical hyperventilation. Low insufflation pressure or gasless LS allows a certain spontaneous respiratory compensation, whereas high insufflation pressure may not due to mechanical respiratory impairment by the abdominal pressure. Hereby, it seems that CO_2_ mainly causes the change of pH, marginally dependent of the insufflation pressure in a second line. To prevent acidic liver flushing during LS, helium may serve as an alternative gas in patients with hepatic restrictions.

Our results support the concern of previous studies that CO_2_-LS bears the potential to cause systemic side effects such as acidic flushing of the liver and the total body [5, 9, 12, 17]. Although, we had demonstrated the detrimental effect of CO_2_-LS on intra-hepatic colorectal tumour growth with an identical experimental setup, direct causality and potential parallels to human LS for neoplasia still need to be demonstrated by further clinical investigation. Nevertheless, surgeons should keep in mind that significant acidic flushing of the liver during CO_2_-LS at least in this experimental model took place and may potentially influence hepatic metabolism and immunological defence with potentially relevant clinical impact in case of pre-existing neoplasia or hepatopathy also in humans.

These mechanisms still need to be elucidated in further detail in a human laparoscopic setup. Meanwhile, LS seems a valid surgical approach.
